# Effects of glycated serum protein, homocysteine, and cystatin-C levels on pregnancy outcomes in patients with gestational diabetes mellitus

**DOI:** 10.12669/pjms.40.4.8988

**Published:** 2024

**Authors:** Yuhua Chen, Chunfang Li, Yurong Lei, Fen Zhang

**Affiliations:** 1 Yuhua Chen, First Ward of Department of Obstetrics and Gynecology, The Yan’an People’s Hospital, 16 Qilipu Street, Yan’an, Shaanxi Province, 716000, P.R. China; 2 Chunfang Li, First Ward of Department of Obstetrics and Gynecology, The Yan’an People’s Hospital, 16 Qilipu Street, Yan’an, Shaanxi Province, 716000, P.R. China; 3 Yurong Lei, First Ward of Department of Obstetrics and Gynecology, The Yan’an People’s Hospital, 16 Qilipu Street, Yan’an, Shaanxi Province, 716000, P.R. China; 4 Fen Zhang, First Ward of Department of Obstetrics and Gynecology, The Yan’an People’s Hospital, 16 Qilipu Street, Yan’an, Shaanxi Province, 716000, P.R. China

**Keywords:** Gestational diabetes mellitus, Glycated serum protein, Homocysteine, Cystatin-C, Pregnancy outcomes

## Abstract

**Objective:**

To explore the effects of serum glycated serum protein (GSP), homocysteine (Hcy) and cystatin-C (Cys-C) levels on pregnancy outcomes in patients with gestational diabetes mellitus (GDM).

**Methods:**

Retrospective selection of 247 pregnant women who underwent normal prenatal examinations in The Yan’an People’s Hospital from January 2020 to May 2022 were included in this retrospective study. Among them, 119 were pregnant women with diabetes (GDM-group) and 128 were pregnant women with normal blood glucose (Normal-group). The levels of serum GSP, HCY, CYS-C, and incidence of adverse pregnancy outcomes were compared between the two groups. The clinical value of levels of serum GSP, Hcy, and Cys-C in predicting adverse pregnancy outcomes were analyzed.

**Results:**

Compared with the Normal-group, the overall incidence of adverse pregnancy outcomes, serum GSP, Hcy, and Cys-C levels in the GDM-group were significantly higher (*p*<0.05). Logistic regression analysis showed that the levels of GSP, Hcy, and Cys-C were independent risk factors for adverse pregnancy outcomes in the GDM-group (*p*<0.05). Receiver operating characteristic (ROC) curve showed that the area under the curve (AUC) for diagnosing adverse pregnancy outcomes in pregnant women with GDM using serum GSP, Hcy, and CysC levels alone were 0.817, 0.843, and 0.775, respectively. The AUC of the three indicators combined was 0.921, indicating that this combination has a good predictive value for diagnosing adverse outcomes in GDM-complicated pregnancies.

**Conclusions:**

GDM is associated with a high risk of adverse pregnancy outcomes. Levels of serum GSP, Hcy, and Cys-C are higher in patients with GDM. The higher the levels of GSP, Hcy, and Cys-C, the greater the risk of adverse pregnancy outcomes. Combining these three indicators can effectively predict maternal pregnancy outcomes.

## INTRODUCTION

Gestational diabetes mellitus (GDM), defined as any manifestation of glucose intolerance with onset or first recognition during pregnancy, is one of the common clinical diseases in pregnant women with an estimated global standardized prevalence of 14%.^1^ Since most patients do not show obvious clinical symptoms, GDM can be found and diagnosed only through blood glucose examination.^2^ GDM disease can increase the risk of macrosomia and negatively affects maternal and neonatal health.^3^ Therefore, more efficient examination and monitoring of patients with GDM, early detection of the condition, and timely treatment and intervention are the key to improving pregnancy outcomes of patients with GDM.^4^

At present, reliable predictive indicators for pregnancy outcomes in patients with GDM in clinical practice are still limited.^5^ Among them, serum glycated serum protein (GSP) is used in combination with other indicators, such as blood glucose, glycated hemoglobin and glycated albumin to assess the glycemic control for diabetic patients.^5^ Cystatin-C (Cys-C) is a sensitive indicator of the renal function of patients, while homocysteine (Hcy) is used to detect cardiovascular and cerebrovascular diseases.^5^,^6^ Serum levels of these indicators have been shown to be associated with maternal and infant complications of patients with GDM, but the results were inconsistent.^5^-^7^ The aim of this study was to explore the predictive value of serum levels of GSP, Hcy, and Cys-C levels for pregnancy outcomes in patients with GDM.

## METHODS

Retrospective selection of 247 pregnant women who underwent normal prenatal examinations in our hospital from January 2020 to May 2022 were included in the study. This included 119 patients with gestational diabetes were selected as GDM group, and 128 pregnant women with normal blood sugar selected as Normal-group. Age of study participants ranged from 22 to 41 years, with an average age of 29.38±3.49 years. The gestational age ranged from 32 weeks to 40 weeks, with an average gestational age of 36 (35, 37) weeks. The Body Mass Index (BMI) before pregnancy was between 21.5 and 28.7kg/m^2^, with an average BMI of 25.3(23.5, 26.5) kg/m^2^.

### Inclusion criteria:

- GDM was diagnosed according to the recommendations by the International Association of the Diabetes and Pregnancy Study Groups.^8^
- Singleton pregnancy.
- Age > 18 years.

### Exclusion criteria:

- Pre-pregnancy diagnosis of diabetes and other metabolic diseases.
- Functional diseases of vital organs such as the heart, liver, and kidneys.
- Concomitant severe infection.
- Those who have taken antihypertensive, hypolipidemic, or hypoglycemic drugs that affect glucose metabolism in the past month.

### Ethical Approval

All procedures involving human participants were done in accordance with the ethical standards of the institutional and/or national research committee(s) and of the Helsinki Declaration (as revised in 2013). Written informed consent was obtained from every patient or their legal guardian. Medical ethics Committee of The Yan’an People’s Hospital approved this study (No.2023-LW-005, Date: 2023-05-08).

Detection of biochemical indicators: For measuring serum levels of GSP, Hcy, and Cys-C, 3 ml of fasting venous blood of all pregnant women was collected in the morning, centrifuged at 4000r/minute (radius of 17 cm) for 10 minutes, and the supernatants were stored at - 80^0^C. GSP levels were detected using nitroblue tetrazolium (NBT) test, Hcy levels were detected using the enzyme circulation method, and Cys-C levels were detected by the latex immunoturbidimetric assay. The kits were purchased from Shanghai mlbio Biotechnology Co., Ltd, and the fully automatic biochemical analyzer was Roche Cobas c702. All procedures were carried out according to the manufacturer’s instructions. Pregnancy outcomes, such as premature delivery, polyhydramnios, macrosomia, fetal distress, and fetal dysplasia were recorded.

### Statistical analysis

SPSS25.0 data software package was used for statistical analysis. The normality of the data was evaluated using the Shapiro-Wilk test. The data of normal distribution were expressed as mean ± standard deviation, and *t*-test was used. The data of non-normal distribution were expressed as median and interquartile interval, and analyzed by Mann-Whitney *U* tests; The counting data was represented by [(n)%], and the inter group data was compared using chi square test. Multiple factor analysis was conducted using logistic regression analysis, and the ROC curve was used to analyze the value of serum GSP, Hcy, and Cys-C levels in predicting pregnancy outcomes. *p*<0.05 indicates a statistically significant difference.

## RESULTS

There was no significant difference in the basic data between the two groups of pregnant women (*p*>0.05) (Table-I). Compared with the Normal-group, the total incidence of adverse pregnancy outcomes in the GDM-group was significantly higher (*p*<0.05) (Table-II). Compared with the Normal-group, the GDM-group had higher levels of serum GSP, Hcy, and Cys-C (*p*<0.05) (Table-III). Logistic regression analysis showed that the levels of GSP, Hcy, and Cys-C were independent risk factors for adverse pregnancy outcomes in the GDM-group (all *p*<0.05) (Table-IV).

**Table-I:** Comparison of Basic Data between Two Groups.

Group	n	Age(years)	Gestational week(weeks)	BMI (kg/m^2^)	Maternity type	

					Primiparous women	Multiparous women
GDM-group	119	29.76±3.87	36(35, 37)	25.1(23.5, 26.5)	96	23
Normal-group	128	29.02±3.07	36(35, 37)	25.35(23.55, 26.5)	97	31
*t/Z/χ^2^*		1.655	-686	-0.861	0.864	
*P*		0.099	0.493	0.389	0.353	

**Table-II:** Comparison of adverse pregnancy outcomes between two groups.

Group	n	Premature birth	Polyhydramnios	Fetal macrosomia	Fetal distress	Fetal dysplasia	Total occurrence rate
GDM-group	119	14(11.8)	9(7.6)	3(2.5)	4(3.4)	2(2.5)	32(26.9)
Normal-group	128	1(0.8)	2(1.6)	0(0.8)	2(1.6)	1(0.8)	6(4.7)
*χ^2^*							23.354
*P*							<0.001

**Table-III:** Comparison of GSP, Hcy, and Cys-C levels between two groups of pregnant women.

Group	n	GSP (μmol/L)	Hcy (μmol/L)	Cys-C (mg/L)
GDM-group	119	295(278, 309)	21(18, 24)	3.2(2.5, 4.0)
Normal-group	128	216(198, 232)	12(11, 14)	0.8(0.5, 1.5)
*Z*		-13.323	-12.783	-12.291
*P*		<0.001	<0.001	<0.001

**Table-IV:** Correlation between the levels of GSP, Hcy, and Cys-C in the GDM-group and adverse pregnancy outcomes.

Factor	B	Wald	P	OR	95%CI
GSP	0.054	10.247	0.001	1.055	1.021~1.090
Hcy	0.424	14.182	<0.001	1.529	1.226~1.907
Cys-C	1.191	9.366	0.002	3.291	1.535~7.056

**Fig.1:**
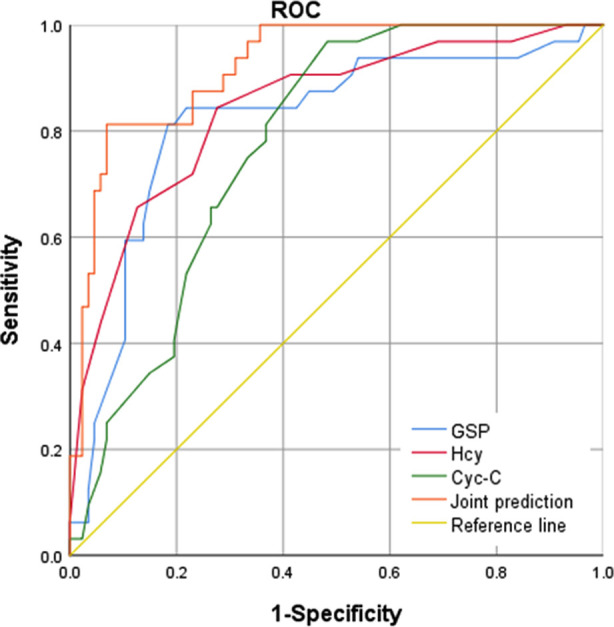
ROC curve of serum GSP, Hcy, and Cys-C levels in predicting adverse pregnancy outcomes.

ROC curve analysis results showed that the area under the curve (AUC) for diagnosing adverse pregnancy outcomes in GDM were 0.817, 0.843, and 0.775 for maternal serum GSP, Hcy, and CysC levels, respectively. The AUC of the three combined tests for diagnosing adverse pregnancy outcomes in GDM was 0.921, indicating a good predictive value (Fig.1).

## DISCUSSION

Our study found that the pregnant women with GDM pregnancy had significantly higher incidence of adverse pregnancy outcomes compared to women with normal pregnancies (P<0.05).

Recent studies have showed that GDM can increase the risk of premature birth in pregnant women, which is in agreement with the results of this study.^9^,^10^ One of the possible mechanisms of this adverse effect is that GDM-associated increase in sugar content in amniotic fluid of pregnant women which may further stimulate amniotic membrane secretion. This, in turn, may lead to an increase in amniotic fluid volume, which is more likely to cause premature rupture of membranes and induce premature birth.^11^ Concurringly, our study found that women in the GDM-group had a higher incidence of both excessive amniotic fluid and premature births compared to the Normal-group. Additionally, studies suggest that GDM may increase the risk of complications such as gestational hypertension and fetal distress. Multiple complications occurring simultaneously may lead to premature termination of pregnancy and iatrogenic premature birth.^12^

In addition, this study showed that the levels of serum GSP, Hcy, and Cys-C in the GDM-group were significantly higher than those in the Normal-group (*p*<0.05), which is consistent with the findings by Jin H^5^ and Gong et al^13^. GSP is one of the common indicators for clinical observation of diabetes control that allows to monitor average glucose levels of the patients over the past two to three weeks. Rising levels of GSP are associated with poorer diabetes control. Multiple studies have found that the combined detection of GSP with hemoglobin A1c (HbA1c) and C-reaction protein (CRP) have a good diagnostic value in the diagnosis of GDM.^14^,^15^ In this study, there was a significant difference in the GSP levels between the two groups of pregnant women, with the GDM-group having significantly higher GSP levels. Our results indicate that GSP can be used to monitor the occurrence and progression of GDM. Hcy is a common clinical marker of cardiovascular and cerebrovascular diseases. An abnormal increase in Hcy is indicative of peripheral blood diseases.^16^ Some studies have found that GDM can be accompanied by increased Hcy, that higher levels of Hcy in pregnant women correlate with the higher risk of developing diabetes after delivery.^17^ Cys-C, is one of the commonly used indicators for clinical evaluation of renal function in patients, and can reflect changes in glomerular filtration rate. Increased Cys-C can indicate renal function decline.^18^ GDM is accompanied by hyperglycemia, which can easily cause hemodynamic abnormalities, increase the risk of hypertension, elevate glomerular pressure, cause glomerular hyperfiltration and damage. Therefore, patients with GDM have increase serum levels of Cys-C.^18^,^19^

In this study, logistic regression analysis showed that the levels of GSP, Hcy, and Cys-C were independent risk factors for adverse pregnancy outcomes in pregnant women with GDM. We propose that an increase in Hcy levels during pregnancy can lead to an increase in asymmetric dimethylarginine levels in the body, causing oxidative stress and exacerbating inflammatory reactions, leading to dysfunction of endothelial cells in the maternal and placental blood vessels, as well as DNA damage. This may cause rapid proliferation of smooth muscle cells and increase the risk of adverse pregnancy outcomes.^16^-^18^ Cys-C is a type of low-molecular-weight non glycosylated protein that can reflect the extent of damage to glomerular filtration function and has certain predictive value for GDM and GDM-related complications.^18^,^20^

Previous studies have confirmed that the combined detection of Hcy and Cys-C can be used to predict adverse pregnancy outcomes in patients with GDM.^20^,^21^ Our study demonstrates that adding GSP to Hcy and Cys-C combination further improve the reliability and sensitivity of the test and increase its predictive value for pregnancy outcomes in patients with GDM.

### Limitations

This is a single center retrospective study with small sample size, which may be prone to selection bias. Secondly, neither group was randomly assigned, and baseline information may be imbalanced and biased, which is also one of the shortcomings of our retrospective study. Thirdly, we only studied serum GSP, Hcy, and Cys-C in this study, more biochemical indicators should be investigated in future studies to further improve the prediction ability of adverse pregnancy outcomes in patients with GDM.

## CONCLUSION

Pregnant patients with GDM have elevated levels of GSP, Hcy, and Cys-C. Combining these three indicators can effectively predict adverse pregnancy outcomes in pregnant women with GDM.

### Authors’ contributions:

**YC:** Conceived and designed the study.

**CL**, **YL** and **FZ:** Collected the data and performed the analysis.

**YC:** Was involved in the writing of the manuscript and is responsible for the integrity of the study.

All authors have read and approved the final manuscript.

## References

[1] Wang H, Li N, Chivese T, Werfalli M, Sun H, Yuen L (2022). IDF Diabetes Atlas Committee Hyperglycaemia in Pregnancy Special Interest Group. IDF Diabetes Atlas:Estimation of Global and Regional Gestational Diabetes Mellitus Prevalence for 2021 by International Association of Diabetes in Pregnancy Study Group's Criteria. Diabetes Res Clin Pract.

[2] Kramer CK, Campbell S, Retnakaran R (2019). Gestational diabetes and the risk of cardiovascular disease in women:a systematic review and meta-analysis. Diabetologia.

[3] Bernea EG, Uyy E, Mihai DA, Ceausu I, Ionescu-Tirgoviste C, Suica VI (2022). New born macrosomia in gestational diabetes mellitus. Exp Ther Med.

[4] Wu YT, Zhang CJ, Mol BW, Kawai A, Li C, Chen L (2021). Early Prediction of Gestational Diabetes Mellitus in the Chinese Population via Advanced Machine Learning. J Clin Endocrinol Metab.

[5] Jin H (2020). Increased levels of glycosylated hemoglobin, microalbuminuria and serum cystatin C predict adverse outcomes in high-risk pregnancies with gestational diabetes mellitus. Exp Ther Med.

[6] Jiang XC, Liang ZD, Chen DL, Jia JP, Hu JR, Hu L (2021). Correlation of Homocysteine, AHSG, CRP with Insulin Resistance, 25-(OH)2-VitD, Blood Lipids in Gestational Diabetes Patients. Clin Lab.

[7] Mascarenhas M, Habeebullah S, Sridhar MG (2014). Revisiting the role of first trimester homocysteine as an index of maternal and fetal outcome. J Pregnancy.

[8] Metzger BE, Gabbe SG, Persson B, Buchanan TA, Catalano PA, Damm P (2010). International association of diabetes and pregnancy study groups recommendations on the diagnosis and classification of hyperglycemia in pregnancy. Diabetes Care.

[9] Di Biase N, Balducci S, Lencioni C, Bertolotto A, Tumminia A, Dodesini AR (2019). Review of general suggestions on physical activity to prevent and treat gestational and pre-existing diabetes during pregnancy and in postpartum. Nutr Metab Cardiovasc Dis.

[10] Ornoy A, Becker M, Weinstein-Fudim L, Ergaz Z (2021). Diabetes during Pregnancy:A Maternal Disease Complicating the Course of Pregnancy with Long-Term Deleterious Effects on the Offspring. A Clinical Review. Int J Mol Sci.

[11] Tian Y, Zhang S, Huang F, Ma L (2021). Comparing the Efficacies of Telemedicine and Standard Prenatal Care on Blood Glucose Control in Women With Gestational Diabetes Mellitus:Randomized Controlled Trial. JMIR Mhealth Uhealth.

[12] Zhang Z, Li J, Hu T, Xu C, Xie N, Chen D (2021). Interventional effect of dietary fiber on blood glucose and pregnancy outcomes in patients with gestational diabetes mellitus. Zhejiang Da Xue Xue Bao Yi Xue Ban.

[13] Gong T, Wang J, Yang M, Shao Y, Liu J, Wu Q (2016). Serum homocysteine level and gestational diabetes mellitus:A meta-analysis. J Diabetes Investig.

[14] Seyhanli Z, Seyhanli A, Aksun S, Pamuk BO (2022). Evaluation of serum Angiopoietin-like protein 2 (ANGPTL-2), Angiopoietin-like protein 8 (ANGPTL-8), and high-sensitivity C-reactive protein (hs-CRP) levels in patients with gestational diabetes mellitus and normoglycemic pregnant women. J Matern Fetal Neonatal Med.

[15] Zheng Y, Deng HY, Qiao ZY, Gong FX (2021). Homocysteine level and gestational diabetes mellitus:a systematic review and meta-analysis. Gynecol Endocrinol.

[16] Liu YH, Lu LP, Yi MH, Shen CY, Lu GQ, Jia J (2022). Study on the correlation between homocysteine-related dietary patterns and gestational diabetes mellitus:a reduced-rank regression analysis study. BMC Pregnancy Childbirth.

[17] Sun YL, Zhou FM, Wang HR (2019). Mechanism of pomegranate ellagic polyphenols reducing insulin resistance on gestational diabetes mellitus rats. Am J Transl Res.

[18] Liu J, Zhang J, Hou MH, Du WX (2022). Clinical efficacy of linagliptin combined with irbesartan in patients with diabetic nephropathy. Pak J Med Sci.

[19] Wojcik M, Zieleniak A, Mac-Marcjanek K, Wozniak LA, Cypryk K (2014). The elevated gene expression level of the A(2B) adenosine receptor is associated with hyperglycemia in women with gestational diabetes mellitus. Diabetes Metab Res Rev.

[20] Li X, Li G, Liu Y, Meng F, Han L, Shao Y (2022). Analysis on the Effect of Metformin Hydrochloride Combined with Insulin Pump for Gestational Diabetes Mellitus. Iran J Public Health.

[21] Zheng J, Xu J, Zhang Y, Zhou N (2020). Effects of insulin combined with metformin on serum cystatin C, homocysteine and maternal and neonatal outcomes in pregnant women with gestational diabetes mellitus. Exp Ther Med.

